# Diverse single-stranded DNA viruses from viral metagenomics on a *cynopterus* bat in China

**DOI:** 10.1016/j.heliyon.2023.e18270

**Published:** 2023-07-17

**Authors:** Yakhouba Kane, Jinping Chen, Linmiao Li, Stéphane Descorps-Declère, Gary Wong, Nicolas Berthet

**Affiliations:** a Viral Hemorrhagic Fevers Research Unit, CAS Key Laboratory of Molecular Virology and Immunology, Institut Pasteur of Shanghai, Chinese Academy of Sciences, Shanghai 200031, China; b University of Chinese Academy of Sciences, Beijing 100049, China; c Guangdong Key Laboratory of Animal Conservation and Resource Utilization, Institute of Zoology, Guangdong Academy of Sciences, Guangzhou 510260, China; d Institut Pasteur, Université Paris Cité, Bioinformatics and Biostatistics Hub, F-75015 Paris, France; e Centre for Microbes, Development and Health, Institut Pasteur of Shanghai, Chinese Academy of Sciences, Discovery and Molecular Characterization of Pathogens Unit, Shanghai 200031, China; f Institut Pasteur, Unité Environnement et Risque Infectieux, Cellule D’Intervention Biologique D’Urgence, 75015 Paris, France; g Institut Pasteur, Université Paris-Cité, Unité Epidémiologie et Physiopathologie des Virus Oncogènes, 75724 Paris, France

**Keywords:** Bat, Cressdnavirus, Genomic characterization, Phylogenetic, China

## Abstract

Bats serve as reservoirs for many emerging viruses. Cressdnaviruses can infect a wide range of animals, including agricultural species, such as pigs, in which porcine circoviruses cause severe gastroenteritis. New cressdnaviruses have also attracted considerable attention recently, due to their involvement with infectious diseases. However, little is known about their host range and many cressdnaviruses remain poorly characterized. We identified and characterized 11 contigs consisting of previously unknown cressdnaviruses from a rectal swab sample of a *Cynopterus* bat collected in Yunnan Province, China, in 2011. Full genomes of two cressdnaviruses (OQ267680, 2069 nt; OQ351951, 2382 nt), and a nearly complete genome for a third (OQ267683, 2361 nt) were obtained. Phylogenetic analyses and the characteristics of these viral genomes suggest a high degree of ssDNA virus diversity. These results shed light on cressdnavirus diversity and the probable role of *Cynopterus* bats as their hosts.

## 1 Introduction

Bats are natural reservoirs for many diverse viruses and some of which are considered emerging viruses. Indeed, they have been confirmed to be the reservoir hosts of SARS-like and MERS-like coronaviruses, and carry the causal agents of rabies and hemorrhagic fevers, such as Marburg virus [1]. As of May 18, 2022, the “DBatVir” database (http://www.mgc.ac.cn/DBatVir/) listed a total of 13,973 known viruses found in bats [2]. These viruses include 504 with single-stranded DNA (ssDNA) genomes, a large proportion of which are classified as eukaryotic, circular, rep-encoding single-stranded (CRESS) DNA viruses (cressdnaviruses).

Cressdnaviruses are non-enveloped viruses belonging to 11 families, including *Circoviridae*. These viruses have relatively short genomes (1500–6500 nt) and are found in many species of mammals, birds, fish, prokaryotes, and plants, and in various environments, including the deep sea. They are extremely divergent, and most remain unclassified. Cressdnaviruses have been associated with many types of diseases caused by different viral species in different hosts, including severe hemorrhagic enteritis in pigs, hepatitis in humans, and severe acute idiopathic chronic diarrhea in rhesus macaques [[3], [4], [5], [6]].

The majority of cressdnaviruses were detected using molecular approaches including polymerase chain reaction and next generation sequencing, and very few were isolated. With the exception of circoviruses (CVs), little is known about the prevalence, transmission, and the impact that cressdnaviruses may have on their hosts. For CVs, transmission occurs principally via contact with contaminated body secretions or excretions, although airborne transmission has also been documented for porcine circovirus [7,8]. Over the last 20 years, porcine CVs have caused major epidemics in the Americas, Europe, and Asia, frequently resulting in the mass culling of pigs [5,7,9].

The growing use of new metagenomic technologies has resulted in the discovery of a spectrum of new cressdnaviruses [10,11]. However, the classification of these viruses has been rendered difficult by an increased number of new cressdnaviruses with unprecedented genome diversity. In this study, we identified and characterized 11 contigs corresponding to new ssDNA viruses, in a rectal swab sample from a greater short-nosed fruit bat (*Cynopterus sphinx*). Two full genomes and one nearly complete genome of new cressdnaviruses (OQ351951, OQ267680, OQ267683) were recovered. Motifs and domains important for cressdnavirus replication, including the nonanucleotide motif, rolling-cycle replication (RCR), and superfamily helicase 3 (SF3) domains, were also identified. Phylogenetic analyses revealed that the identified ssDNA viruses were unclassified cressdnaviruses belonging to the class *Arfiviricetes.*

## 2 Materials and methods

### 2.1 Sample processing and sequencing

As part of a previous investigation based on high-throughput sequencing approach (unpublished work), an analysis of *Cynopterus* bat samples collected in Yunnan Province in 2011 identified two contigs of 450 nt and 150 nt related to cressdnaviruses in one rectal swab sample. We used a method combining previously described protocols for viral metagenomics to amplify the entire genomes of these suspected cressdnaviruses [[12], [13], [14]]. Two aliquots of the sample in question were retrieved from the −80 °C freezer and centrifuged at 1800×*g* and 4 °C, for 15 min. Eukaryotic and bacterial cell debris was removed by passing the supernatants through a filter with 0.45 μm pores. The first aliquot (the untreated sample) was used for nucleic acid extracted with the RNA Easy Fast Tissue/Cell kit (TIANGEN). In the second aliquot (treated sample), the unprotected nucleic acids were digested with a cocktail of enzymes — 15 U Turbo DNase, 20 U Benzonase, 20 U RNase One, in 10*x* Turbo DNase buffer — and 30 mM EDTA was then added and incubated with the mixture for 30 min to stop the enzymatic reaction [12,13]. The nucleic acids obtained from the two sample aliquots were then subjected to reverse transcription with the ProtoScript II First-Strand cDNA Synthesis Kit (New England Biolabs), followed by second-strand synthesis with the NEBNext Ultra II Non-Directional RNA Second-Strand Synthesis Module (New England Biolabs). Two libraries were constructed with the NEBNext Ultra II library preparation kit, in accordance with the protocol recommended by the manufacturer (Illumina) for use with FFPE RNA. The libraries were pair end-sequenced on an Illumina Novogene 6000 device.

### 2.2 Sequence data analysis

Raw reads were processed with fastp tools to remove adaptor contaminants and reads of low quality, as previously described [15]. The generated clean reads were further *de novo* assembled into contigs with SPAdes v. 3.15.4 [16]. Contig assemblies were compared against the National Center for Biotechnology Information non-redundant protein database (NCBI-nr) using DIAMOND v2.0.15.153, with e-value cutoffs of ≥1E−10 and ≥1E−4 [17]. Contigs related to unclassified viruses with circular genomes and cressdnaviruses were selected and putative open reading frames (ORFs) were detected using getorf (https://www.bioinformatics.nl/cgi-bin/emboss/getorf) and NCBI ORF Finder (https://www.ncbi.nlm.nih.gov/orffinder/) [18,19]. BLAST analysis was performed on the NCBI-nr protein database to confirm the identity of the ORFs. Metagenomics reads from the two sample aliquots were mapped to their corresponding viral contigs or genomes with minimap v2.1 [20]. Only contigs with a length of more than 500 bp were annotated according to the best hit following taxonomic analyses.

### 2.3 Phylogenetic analyses

Amino acid (aa) sequences of the replicase (Rep) protein of cressdnaviruses were downloaded from GeneBank to compile two datasets: one for the family *Circoviridae*, and another for the order *Cressdnavircota* (Supplementary data 1 & 2). Multiple sequence alignment was generated with MAFFT version 7 [21]. Phylogenetic analyses were performed with the maximum likelihood method implemented in IQ-TREE multicore version 2.0.3 [22]. The best-fit model was determined with ModelFinder (-m MF) and nodes were supported by ultrafast bootstrapping, with a total of 1000 replicates retained [23]. Phylogenetic analyses were performed for the cressdnaviruses for which the complete genome was obtained.

## 3 Results

### 3.1 Metagenomics results

**Fig. 1:**
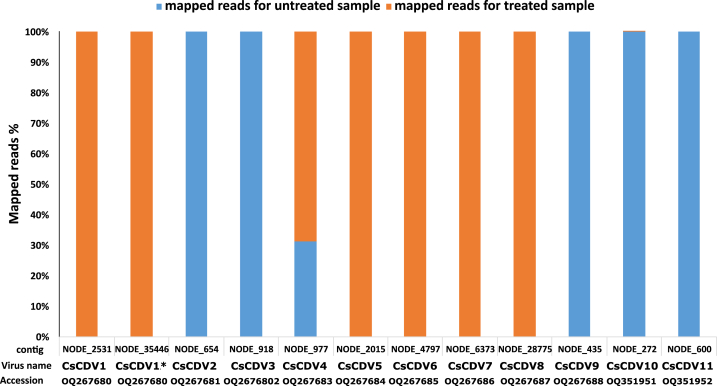
Reads mapped (%) onto the selected cressdnavirus contigs. CsCDV: *Cynopterus sphinx* circular DNA virus. * indicates partial Cap contig of CsCDV1.

The sequencing of the two libraries revealed a total of 206, 555, 880 (untreated sample) and 58, 018, 426 (treated sample) raw reads and the number of filtered reads accounted for 64, 726, 140 (untreated sample) and 6,644,640 (treated sample) reads. Following read filtering and adaptor contaminants removal, *de novo* assembly of clean reads from the two libraries generated a total of 119 cressdnavirus contigs (73 contigs for the untreated sample, 46 contigs for the treated sample) and 8 unclassified circular ssDNA virus contigs (4 contigs for the untreated sample, 4 contigs for the treated sample) (Supplementary data 3 & 4). Over half of these contigs corresponded to new unclassified cressdnaviruses. Eleven contigs (>500 nt) corresponding to new cressdnaviruses were retained for this study. Read mapping onto these cressdnavirus contigs revealed that only one contig (OQ267683) was recovered from both libraries (Fig. 1) (Supplementary data 5). This suggests that different approaches to library preparation for the metagenomics of cressdnaviruses may have different implications for the results. Further studies with a larger number of samples are required to investigate this aspect. The GenBank accession numbers of the cressdnaviruses identified in this study are as follows: OQ267680-OQ267688, OQ351951, OQ351952 (Table 1).

**Table 1:** List of putative viruses discovered in this study. Viral genomes/contigs were compared with NCBI-nr using DIAMOND BLASTx.

Taxon	Virus/contig name (GenBank accession no.)	Genome/Contig (bp)	Best hit NCBI-nr	Similarity (%)	E-value	Host
***Cressdnavircota***	*Cynopterus sphinx* circular DNA virus 1 (OQ267680)	2069	*Fringilla montifringilla Circoviridae* sp. (QTE03314.1)	100 (Rep)	1.12E-225	Bird (*Fringilla montifringilla*)
				90.8 (Cap)	8E-180	
***Cressdnavircota***	*Cynopterus sphinx* circular DNA virus 2 (OQ267681)	1017	*Circovirus*-like genome DCCV-2 (YP_009259705.1)	46.7 (Rep)	5.58E-51	Environmental sample (freshwater lake)
***Cressdnavircota***	*Cynopterus sphinx* circular DNA virus 3 (OQ267682)	845	*Circoviridae* sp. (AXH74395.1)	48.1 (Cap)	1.10E-60	Fish (minnow)
***Cressdnavircota***	*Cynopterus sphinx* circular DNA virus 4 (OQ267683)	2361	Uncultured virus (AUM61988)	87.97 (Rep)	2E-97	Mammal (Manatee Springs)
			Clinch CRESS virus 1 (QNL09587.1)	51.9 (Cap)	3E-115	
***Cressdnavircota***	*Cynopterus sphinx* circular DNA virus 5 (OQ267684)	1764	Lake Sarah-associated circular virus-28(YP_009237569.1)	36.5 (Cap)	1.17E-57	Snail (*Potamopyrgus antipodarum*)
***Cressdnavircota***	*Cynopterus sphinx* circular DNA virus 6 (OQ267685)	1224	*Nandayus nenday* CRESS-DNA-virus sp. (QTE03394.1)	37.6 (Rep)	9.22E-43	Mammal (Manatee Springs)
***Cressdnavircota***	*Cynopterus sphinx* circular DNA virus 7 (OQ267686)	1079	CRESS viruses (QJI53686.1)	35.4 (Rep)	3.48E-12	Bird (*Aratinga nenday*)
***Cressdnavircota***	*Cynopterus sphinx* circular DNA virus 8 (OQ267687)	523	CRESS viruses (AXH73775.1)	48.2 (Rep)	3.37E-18	Fish (minnow)
***Cressdnavircota***	*Cynopterus sphinx* circular DNA virus 9 (OQ267688)	1387	Lake Sarah-associated circular virus-28(YP_009237569.1)	35.71 (Cap)	3.00E-55	Snail (*Potamopyrgus antipodarum*)
***Cressdnavircota***	*Cynopterus sphinx* circular DNA virus 10 (OQ351951)	2382	*Cressdnavircota* sp. (WAE43075.1)	39.76% (Cap)	1E-91	Environmental sample (soil)
			Uncultured marine virus (AGA18411.1)	62.88% (Rep)	2E-118	Environmental sample (Saanich inlet)
***Cressdnavircota***	*Cynopterus sphinx* circular DNA virus 11 (OQ351952)	1069	CRESS virus sp. (AWW06082.1)	37.61% (Cap)	4E-41	Amphibian (axolotl tissue)

### 3.2 Genomic characterization

**Fig. 2:**
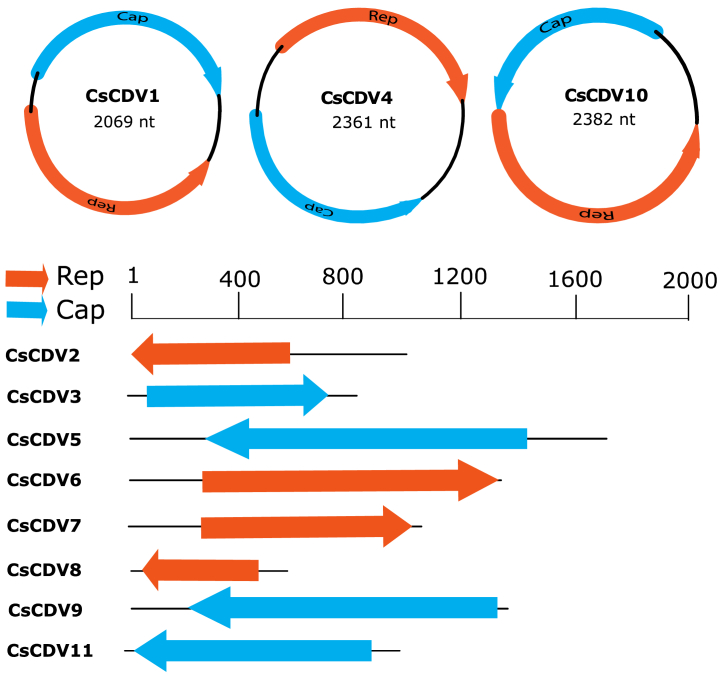
Representation of the cressdnavirus genomes/contigs identified in this study. Genomes or contigs with two ORFs are circularized, and contigs with one ORF are represented in linear form.

The genome organization of the cressdnaviruses characterized in this study is shown in Fig. 2. We obtained full genome sequences for two cressdnaviruses. The first genome corresponds to a cressdnavirus named *Cynopterus sphinx* circular DNA virus 1 (CsCDV1) (OQ267680). It is 2069 nt long and consists of two ORFs: a Rep of 933 nt (311 aa) and a capsid (Cap) of 876 nt (292 aa). The second complete genome, corresponding to *Cynopterus sphinx* circular DNA virus 10 (CsCDV10), is a 2382 nt sequence from another new cressdnavirus (OQ351951). The putative Rep 1257 nt (419 aa) and Cap 792 nt (264 aa) sequences are present in the reverse orientation in this genome. An almost complete genome (2361 nt) sequence for another new cressdnavirus, *Cynopterus* circular DNA virus 4 (CsCDV4, OQ267683) was also identified. Its ORF1 corresponds to a long Cap sequence of 1179 nt (393 aa), followed by a second ORF identified as a partial Rep of 519 nt (173 aa). According to the classification of ssDNA viruses on the basis of genomic features, both CsCDV1 and CsCDV4 are type II cressdnaviruses (Rosario et al., 2012). Putative nonanucleotide motifs were identified only for CsCDV3 (TAATATTAC) and CsCDV5 (TAGTATTAC). The number and orientation of ORFs, the presence of the nonanucleotide motif, and the genome type are summarized below (Table 2).

**Table 2:** Characteristics of the cressdnavirus genomes described in this study.

Virus name	Genome/contig length	Genome type	Nonanucleotide motif	Rep (start-stop)	Cap (start-stop)	Accession
CsCDV1	2069 nt	II	NA	147–1082 ^(−)^	1200–2060^(+)^	OQ267680
CsCDV2	1017 nt	NA	NA	1–576^(−)^	NA	OQ267681
CsCDV3	845 nt	NA	TAATATTAC	NA	78–737	OQ267682
CsCDV4	2361 nt	II	NA	1885–2361^(+)^	478–1659^(−)^	OQ267683
CsCDV5	1764 nt	NA	TAGTATTAC	NA	339–1391^(+)^	OQ267684
CsCDV6	1224 nt	NA	NA	367–1224^(+)^	NA	OQ267685
CsCDV7	1079 nt	NA	NA	380–1078^(+)^	NA	OQ267686
CsCDV8	523 nt	NA	NA	3–428^(−)^	NA	OQ267687
CsCDV9	1387 nt	NA	NA	NA	284–1384^(−)^	OQ267688
CsCDV10	2382 nt	NA	NA	1259–2053^(−)^	3–1262^(−)^	OQ351951
CsCDV11	1069 nt	NA	NA	NA	1–900^(−)^	OQ351952

^(+)^ Sense orientation; ^(−)^ Inverse orientation.

**Fig. 3:**
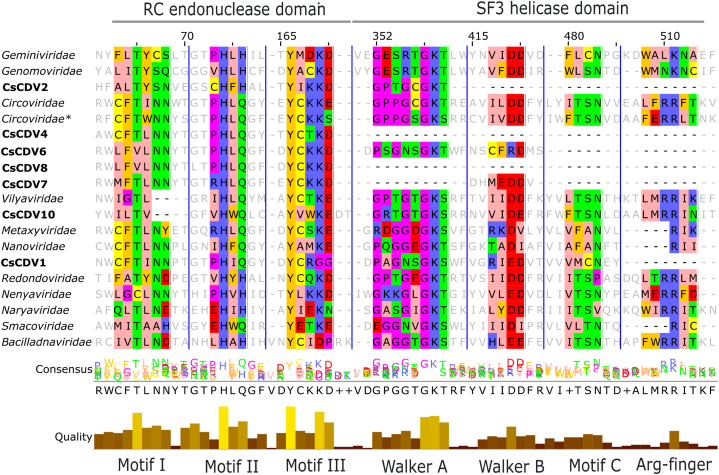
Motifs identified in the rolling-cycle replication and superfamily helicase 3 domains of the cressdnaviruses discovered in this study (Supplementary data 6). Rep proteins of representative members of established cressdnavirus families were used, and the sequences from this study are in bold typeface. The residues are colored according to their physicochemical properties (positive, blue; negative, red; hydrophobic, green; cysteine, yellow; aromatic, orange; aliphatic/hydrophobic, pale pink; conformationally special, fuchsia pink) (Waterhouse et al., 2009). * genus *Cyclovirus*.

By scanning the Rep protein, we detected motifs and domains characteristic of cressdnaviruses, including the RC endonuclease and SH3 helicase domains (Fig. 3). Over the entire length of the genome, CsCDV1 displayed a high degree of nucleotide identity to *Fringilla montifringilla Circoviridae* sp. (MW182726), Reagent-associated CRESS-like virus 2 (MZ824236), and *Circoviridae* sp. (MW202791), which were previously identified in China, Australia, and the US, respectively. The main differences include the presence of 14 deletions in the Cap protein sequence and the arrangement of the ORFs in the genome of CsCDV1. Comparisons of the aa sequences of the Rep protein revealed that CsCDV4 and CsCDV10 were 87.97% and 62.88% identical, respectively, to unclassified cressdnaviruses recovered from environmental samples (AUM61988) and axolotl tissues (AGA18411.1), respectively.

Two other cressdnavirus contigs, CsCDV2 (1017 nt, OQ267681) and CsCDV3 (845 nt, OQ267682), were identified as partial genomes with a high level of divergence, displaying 46.7% (Rep) and 48.1% (Cap) aa similarity to other unclassified circoviruses (YP_009,259,705, AXH74395, AXH75561) (Table 1). Contigs corresponding to other new cressdnaviruses (OQ267683, OQ267685, OQ267686, OQ267687, OQ351952) encoded aa sequences with the lowest degree of similarity, ranging from 35.4 to 52.2% aa sequence similarity to previous unclassified cressdnaviruses (QJI53686, QTE03394, AXH73775, QNL09587). BLAST analysis also uncovered two contigs — CsCDV5 (OQ267684, 1764 nt) CsCDV9 (OQ267688, 1387 nt) — with a low level of aa sequence similarity (∼36%) to known cressdnaviruses (Table 1). The closest hit to these contigs was Lake Sarah-associated circular virus-28 (YP_009,237,569), a circular ssDNA virus not assigned to any known viral taxon.

### 3.3 Phylogenetic analysis of the identified cressdnaviruses

**Fig. 4:**
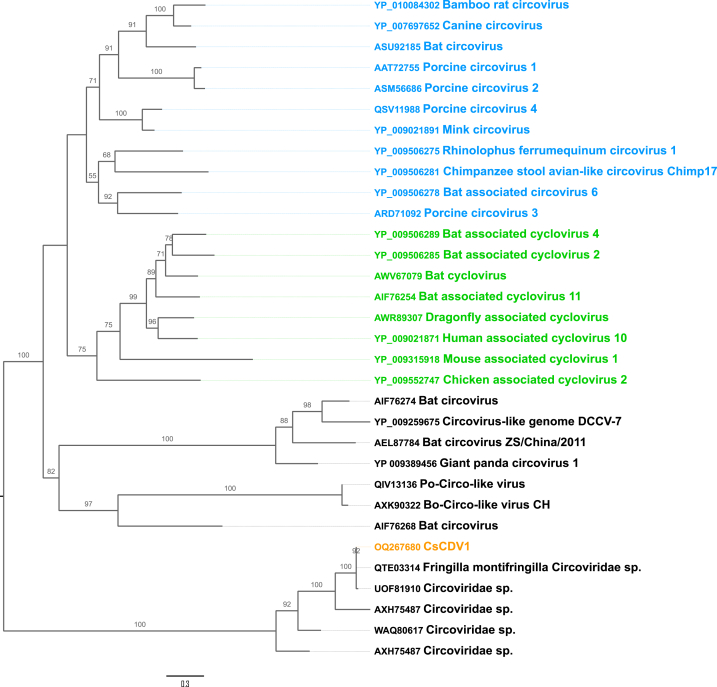
Maximum likelihood (ML) phylogenetic tree for CV Rep. Blue and green correspond to the genera *Circovirus* and *Cyclovirus*, respectively, and the remaining clades contain unclassified circoviruses or circo-like viruses. The cressdnavirus from this study is shown in orange. The tree was inferred with IQ-TREE multicore version 2.0.3, using the Q. pfam + I + G4 substitution model following alignment with MAFFT version 7 (Chernomor et al., 2016; Katoh et al., 2019). The phylogenetic tree was rooted at the midpoint and branches were supported by Ultrafast bootstrapping with 1000 replicates. The scale bar represents the number of substitutions per site.

**Fig. 5:**
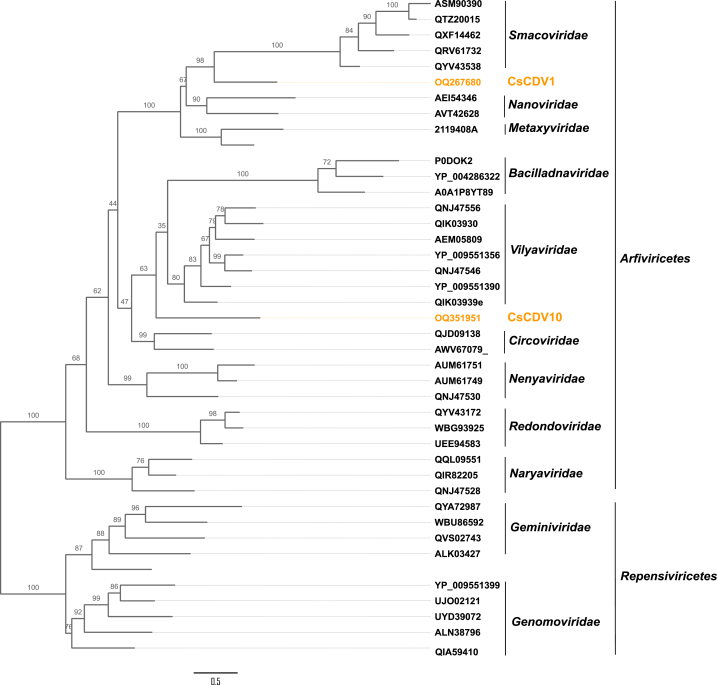
Maximum likelihood (ML) phylogenetic tree for Rep aa sequences from representative members of known cressdnavirus families. The cressdnaviruses obtained in this study are shown in orange. The tree was inferred with IQ-TREE multicore version 2.0.3, using the Q. pfam + F + R4 substitution model following alignment with MAFFT version 7. The phylogenetic tree was supported by Ultrafast bootstrapping with 1000 replicates (Chernomor et al., 2016; Katoh et al., 2019). The scale bar represents the number of substitutions per site.

Phylogenetic analyses based on the Rep protein sequences of CVs revealed that CsCDV1 clustered away from well-established CV genera (Fig. 4). CsCDV1 belonged to the same clade as a circovirus detected in a *Fringilla* bird in China (MW182726). Other close relatives of CsCDV1 included several sequences labeled as circovirus sequences from different vertebrates (UOF81910, AXH75487, WAQ80617). We included CsCDV1 in a second phylogenetic analysis based on Rep aa sequences from established cressdnavirus families (Fig. 5). Both CsCDV1 and CsCDV10 were found to diverge from these cressdnavirus families, but to belong to the class *Arfiviricetes*, of the phylum *Cressdnavircota*.

## 4 Discussion

The objective of this study was to identify and characterize cressdnaviruses in a *Cynopterus sphinx* bat rectal swab sample collected in Yunnan Province in 2011. *Cynopterus sphinx* is a megabat species in the “of least concern” category of the International Union for the Conservation of Nature [24]. It breeds twice per year and is found principally in Sri Lanka, India, Bangladesh, and mainland Southeast Asia, including southern China. This bat species has not yet been implicated as a reservoir for any particular emerging virus dangerous to humans, but its geographic distribution overlaps an area in which many emerging viruses have been identified from other animal species [25].

Over the last few years, the widespread use of high-throughput sequencing technologies has resulted in the discovery of numerous new cressdnaviruses, many of which remain poorly characterized and unclassified. With the exception of CsCDV1, the cressdnaviruses characterized here displayed a low level of aa sequence similarity to other unclassified cressdnaviruses isolated from various hosts, including mammals, birds, fish, and even environmental samples. These findings indicate that much remains unknown about the host range of cressdnavirus taxa.

The family *Circoviridae* contains two well-established genera — *Circovirus* and *Cyclovirus* — with short genomes (1600–2200 bp), typically containing two ORFs. Phylogenetic analysis of the Rep proteins of CVs showed that CsCDV1 clustered outside of the two circovirus genera, together with other unclassified circoviruses. Given the significant divergence between the clade to which CsCDV1 belongs and other circoviruses, we further investigated CsCDV1 by generating a second phylogenetic tree based on the Rep proteins of cressdnavirus families. Both CsCDV1 and CsCDV10 were considered unclassified cressdnaviruses based on this analysis. Thus, although the BLAST results suggested that CsCDV1 was a circovirus, phylogenetic analyses suggested that CsCDV1 might be an unclassified cressdnavirus instead.

Several metagenomics investigations have revealed the existence of a huge diversity of ssDNA viruses with novel genome architectures [10,26,27]. Some of these viruses including satellite viruses, nanoviruses, and several non-characterized ssDNA viruses with genomes containing a single ORF have been identified [28]. Here, we identified sequences related to divergent cressdnaviruses, and most (6/8) of the contigs related to these new cressdnaviruses contained a single ORF (3 Rep and 3 Cap) (Table 2). BLAST analyses identified circovirus sequences as the best hits for CsCDV2 and CsCDV3, but we did not include these viruses in the phylogenetic analyses due to the shortness of their genomes. Overall, the level of diversity of the cressdnaviruses identified in this study suggests a need for further taxonomic efforts to establish new cressdnavirus taxa.

The nonanucleotide motif, RCR, and SF3 domains are conserved in many ssDNA viral taxa and are known to be important for replication. The RCR and SF3 domain motifs found in this study differed substantially from those found in cressdnavirus families. The high diversity of these cressdnaviruses at these motifs and domains levels suggests complex evolutionary roots. Also, the question whether these cressdnaviruses belong to the same original host cannot be solved here. However, it is well-known that bats have the potential to be persistently infected with different viruses, and the rapid evolution of ssDNA virus genomes, with mutation rates similar to those of dsRNA viruses can facilitate the occurrence of such phenomenon in nature [29].

The primary limitation of this study is the analysis of a single sample from a *Cynopterus* bat. Studies on a larger number of samples will be required to determine conclusively whether *Cynopterus* bats are genuine hosts of the cressdnaviruses identified in this study. Another feature of this study was the use of an RNA extraction kit to isolate nucleic acids. RNA isolation kits like that used here have been used in previous metagenomics studies of ssDNA viruses [12,27]. However, most of the spin columns widely used for RNA extraction can retain ssDNA, the amounts of ssDNA recovered depending on many factors, including the buffer, and the size and concentration of the ssDNA present in the sample. It also remains unknown whether the cressdnaviruses identified in this study are of dietary origin or pathogenic to *Cynopterus sphinx.* Further investigations with a larger number of samples will be required to address this question.

In recent years, cressdnaviruses, particularly CVs/circo-like viruses, have been associated with a broad range of diseases in mammals. Given the potential threat posed to agricultural livestock and the established role of bats as hosts or reservoirs of emerging zoonotic viruses of significant medical interest, there is a need for further studies to determine the role of bats in intra- and interspecies transmission of cressdnaviruses, and, more importantly, the dynamics and molecular basis of bat host-cressdnavirus interactions. It will also be of interest to determine, at the population level, whether these viruses can act as a “marker” for potential virus transfer between and within bat individuals/species. Furthermore, there is currently a substantial gap in our knowledge relating to virome surveys in certain bat species. In the case of *Cynopterus sphinx*, such studies are almost non-existent. Future surveys targeting neglected bat families of microbial and ecological interest will therefore be invaluable, for understanding the role that these animals play in the maintenance and the transmission of pathogens in nature.

## 5 Conclusions

We report here the identification and description of new divergent cressdnaviruses from a single rectal swab sample from a *Cynopterus* bat in China. We identified 11 contigs corresponding to new unclassified cressdnaviruses, and recovered two whole genomes and one nearly complete genome. Taxonomic and phylogenetic analyses revealed significant divergence of these new viruses from established cressdnavirus families. These findings suggest that the *Cynopterus* bat is a potential host for cressdnaviruses.

## Author contribution statement

Yakhouba Kane: performed the experiments, analyzed and interpreted the data, wrote the paper.

Jinping Chen: contributed to reagents, materials, analysis tools or data.

Linmiao Li: contributed to reagents, materials, analysis tools or data.

Stéphane Descorps-Declere: analyzed and interpreted the data.

Gary Wong: conceived and designed the experiments, contributed to reagents, materials, analysis tools or data, wrote the paper.

Nicolas Berthet: conceived and designed the experiments, contributed to reagents, materials, analysis tools or data, wrote the paper.

## Data availability statement

Data associated with this study has been deposited at GenBank: OQ267680-OQ267688, OQ351951, OQ351952.

## Additional information

Supplementary content related to this article has been published online at [URL].

## Funding

This project was supported by the Ministry of Science and Technology of the People's Republic of China (Grant No. 2021YFC0863400), the Alliance of International Scientific Organizations (Grant No. ANSO-CR-SP-2020-02), the Shanghai Municipal Science and Technology Major Project (Grant No. 2019SHZDZX02), G4 funding from Institut Pasteur, Fondation Merieux and Chinese Academy of Sciences to G.W., and the International Affairs Department of the Institut Pasteur of Paris, and the 2021 Project of Guangdong Forestry Bureau to J.C. Y.K. is supported by the CAS-TWAS Fellowship for International Doctoral Students.

## Declaration of competing interest

The authors declare that they have no known competing financial interests or personal relationships that could have appeared to influence the work reported in this paper.
